# In situ rumen degradation characteristics and bacterial colonization of whole cottonseed, cottonseed hull and cottonseed meal with different gossypol content

**DOI:** 10.1186/s13568-021-01244-2

**Published:** 2021-06-22

**Authors:** Wei-Kang Wang, Yan-Lu Wang, Wen-Juan Li, Qi-Chao Wu, Kai-Lun Yang, Sheng-Li Li, Hong-Jian Yang

**Affiliations:** 1 State Key Laboratory of Animal Nutrition, College of Animal Science and Technology, China Agricultural University, Beijing, 100193 China; 2 College of Animal Sciences, Xinjiang Agricultural University, Urumuqi, 830052 China

**Keywords:** Attached bacteria, Cotton by-products, Gossypol, In situ degradation

## Abstract

Regarding whole cottonseed (WCS), cottonseed meal (CSM), and cottonseed hull (CSH), in situ rumen incubation was applied to determine their nutrient and gossypol degradation characteristics and bacterial colonization profile in lactating Holstein cows. Nylon bags containing the cotton by-products were incubated for 0, 6, 12, 24, 36, 48 and 72 h in the rumen, respectively. The relationship between nutrient degradability and free gossypol (FG) content were examined, and the differences in the composition and inferred gene function of the colonized microbiota were studied. As a result, CSM presented highest effective degradability of dry matter, neutral detergent fibre and acid detergent fibre, but the highest effective degradability of crude protein was found in WCS. Free gossypol disappearance rate increased significantly in the first 6 h, and it reached approximately 94% at 72 h of incubation among all samples. The level of FG did not affect nutrient degradability of cotton by-products. Significant differences were noted in attached bacterial community structure among cotton by-products after 24 h rumen incubation. Among the most abundant taxa at genus level, a greater abundance of *Cercis gigantea* and *Succiniclasticum* was observed in WCS samples, whereas the CSH and CSM samples contained a greater proportion of *Prevotella 1* and *Rikenellaceae RC9 gut group*. The redundancy analysis revealed that the level of neutral detergent fibre, ether extract, and FG in cotton by-products were significantly positive related with the composition of the attached bacteria. Collectively, our results revealed the dynamics of degradation characteristics, and the difference in the composition of bacterial colonization. These findings are of importance for the targeted improvement of cotton by-products nutrient use efficiency in ruminants and further understanding of the gossypol degradation mechanism in the rumen.

**Supplementary Information:**

The online version contains supplementary material available at 10.1186/s13568-021-01244-2.

## Key points

- FG had no significant effect on nutrient degradability of cotton by-products.
- NDF, EE, and FG content in cotton by-products were significantly related with the composition of the attached microbiota.
- Genes of attached bacteria relating to energy metabolism were more abundant in WCS which presents higher FG content.

## Introduction

Rumen microbiome is a highly diverse collection of obligate anaerobic microorganisms including fungi, protozoa, bacteria, and archaea (Russell and Rychlik 2001). Particle-associated bacteria account for 70–80% of rumen microbial matter (Craig et al. 1987), and bacterial attachment is an important instrumental in the process of microbial degradation of dietary plant material (McAllister et al. 1994). Thus, attached bacteria are believed to be the dominant domain, making the greatest contribution to degradation and conversion of feeds in the rumen (Kim et al. 2011). Cotton by-products have been extensively accepted as energy or protein source in diets of ruminant animals though they contain toxic free gossypol (FG), a polyphenolic compound produced by cotton (*Gossypium* sp.) (Santos et al. 2002), the toxicity of FG is mainly because of its active hydroxyl and aldehyde groups. The main clinical signs of excessive gossypol intake are weakness, apathy, impaired body weight gain, respiratory distress, and even death within a short period in various ruminant animals (Risco et al. 1992; Zelski et al. 1995; Alexander et al. 2008). We believed that the study of attached bacteria on cotton by-products should contribute to the further understanding of gossypol degradation and transformation mechanism in the rumen.

In situ nylon bag technique is a widely adopted procedure to characterize the dynamics of nutrient degradation of feedstuffs in the rumen. Bo et al. (2012) determined the degradation characteristic of cottonseed hulls processed in different ways with in situ rumen incubation. Liu et al. (2016) and Qian et al. (2019) revealed the dynamics of colonization by bacterial communities attached to cottonseed hull and other feeds in the rumen by in situ nylon bag technique. These researches suggesting that the in situ nylon bag technique was a useful method to determine the interaction of rumen microbial diversity and feeds degradation characteristics. Although ruminants in comparison with monogastric animals are believed to have high tolerance to FG toxicity due to microbial fermentation in the rumen, there is no research have been reported on the relationship between the chemical composition and attached bacteria of cotton by-products in the rumen.

In the present study, representative samples of whole cottonseed (WCS), cottonseed meal (CSM) and cottonseed hull (CSH) with different FG content were chosen as experimental materials, and the objective was to determine the effect of FG content on the rumen degradation of cotton by-products as well as the association between chemical composition and solid attached bacteria diversity using an in situ rumen incubation method.

## Materials and methods

### Cotton by-products

Representative cotton by-products were collected from the northern region of Tianshan Mountain in the Xinjiang Uygur Autonomous Region of China for the production of long lint cotton (*Gossypium* spp.), including three WCS samples, three lint CSH samples and two CSM samples. All samples were dried at 65 °C for 24 h in a forced air oven, ground to pass through a 2.00—mm screen, stored prior to chemical analyses and in situ degradation trials.

### Animals

Three mature, rumen-cannulated, lactating Holstein cows (average body weight: 502 ± 25 kg, 151 days in milk and 36 kg/day of milk yield at the beginning of the study) were used in this study. Cows were individually housed in three stalls with good ventilation and were fed 25 kg of dry matter (DM) (110 g/kg of imported Alfalfa, 490 g/kg of silage maize, 140 g/kg of pressed corn, and 260 g/kg of mixture) per animal per day, and free access to drinking water. The cows were fed at 0900 and 1500 h, and milked three times daily. All animal experimental procedures were conducted in accordance with the In stitu-tional Animal Care Committee and animal welfare guidelines of China Agricultural University (CAU20171014-1).

### In situ rumen incubation and sampling procedure

Regarding each cotton by-product sample, approximately 5 g of the ground samples were weighed into individual in situ nylon bags (10 × 20 cm; pore size = 50 μm), bags containing the samples were introduced into rumen for periods of 0, 6, 12, 24, 36, 48 and 72 h. Duplicate bags were incubated in each animal for each incubation time. All bags of each period were placed in the rumen at 0800 h of the morning simultaneously. Three replications were performed for periods of 0, 6, 12 and 24 h of each sample in each animal, and four replications were performed for periods of 36, 48 and 72 h.

After removal of all bags containing samples of each incubated time from the rumen, six bags (two bags per sample per cow) were rinsed and manipulated in tap water until the water ran clear, then squeezed by hand to remove excess water and dried at 65℃ for 24 h for in situ dry matter disappearance rate analysis, and pool six bags together for nutritional ingredient and FG degradation analysis. The remaining bags of per sample per cow were squeezed by hand to remove loosely attached microbes, pooled and transferred the residue sample to the 2 mL quick freezing pipe for the future research of solid-attached bacteria. Samples were ground in liquid nitrogen quickly, and transported to the laboratory for storage at − 80 °C until DNA extraction.

### Chemical analysis

For the representative cottonseed by-product samples and the residues used in the trial, DM, crude protein (CP) and ether extract (EE) were determined according to AOAC methods (AOAC 1999), and neutral detergent fibre (NDF) (assayed with sodium sulphite and a heat stable amylase) and acid detergent fibre (ADF) were determined were analysed following the method of Van Soest et al. (1991).

### Determination of free gossypol

Cotton by-products and residual samples in the nylon bags (0.15 g) in 1.5 mL acetone were ultrasound 30 min at 40 ℃, centrifuged with the speed of 1000 × g at 25 ℃ for 10 min, collected the supernatant, repeat the above processes three times, the extraction were combined and filtrated with 0.45 μm microporous, rotary evaporated, dissolved by acetonitrile—0.2% phosphoric acid solution and fixed capacity to 2.5 mL.

The content of FG was quantified by high-performance liquid chromatography (HPLC) with a Wufeng analytical instrument (Wufeng Co., Ltd, Shanghai, China) consisting of LC-P100PLUS pump, LC-UV100PULS UV detector and LC-CO100PLUS column heater. The analytical column was a symmetry reversed-phase C18 column (250 × 4.6 mm, 5 μm, pH 2–8, Waters, Milford, MA, USA). The mobile phase was 85:15 (v/v) acetonitrile—0.2% phosphoric acid solution at a flow rate of 1 mL/min. Injections were 20 μL, and the FG was detected at 235 nm.

100 mg standard gossypol was dissolved into 1 mL mobile phase, and diluted into 100, 50, 25, 12.5, 6.25, 3.125, 1.55, 0.78, 0.36, 0.18, 0.09 μg/mL by mobile phase. The standard curve was obtained by linear regression of peak area Y and gossypol concentration X under optimal chromatographic conditions as noted above.

### Biometric analysis

The ruminal disappearance (Y) of DM, CP, NDF and ADF at time (t) was fitted to an exponential model by an iterative regression analysis (Ørskov and McDonald 1979) using the GLM procedure of SAS (1999). The model is described by Eq. (1):

1 $${\text{Y }} = {\text{ a}} + {\text{b}} \times \left( {{1} - {\text{e}}^{{ - {\text{c}} \times {\text{t}}}} } \right)$$

where ‘e’ is the base of a natural logarithm; the constant “a” represents the soluble and very rapidly degradable component and “b” represents the insoluble, but potentially degradable component, which has a constant fractional degradation rate (c) per unit time. The effective degradability (ED) of DM, CP, NDF and ADF were then estimated using Eq. (2) (Ørskov and McDonald 1979):

2 $${\text{ED}} = {\text{a}} + \frac{{{\text{b}} \times {\text{c}}}}{c + k}.$$

where ‘k’ refers to the fractional outflow rate. A value of 0.06/h was assumed for k, as suggested in the literature (INRA 1988).

### Collection of solid attached microbiota and DNA extraction

The solid attached bacteria were collected according to the method of Larue et al. (2005). Total microbial genomic DNA was extracted using OMEGA Stool DNA Isolation Kit (MoBio Laboratories, Carlsbad, CA) following the manual. The DNA quality and concentration were monitored using NanoDrop 1000 spectrophotometry (Thermo Scientific, Waltham, EUA).

### PCR amplification, High throughput sequencing and sequencing data processing

The V4 hypervariable region of bacterial 16S rRNA gene were amplified with the primers 515F (5’-GTGCCAGCMGCCGCGGTAA-3’) and 806R (5’-GGACTACHVGGGTWTCTAAT-3’) (Evans et al. 2014). The PCR was carried out on a Mastercycler Gradient (Eppendorf, Germany) using 25 μL reaction volumes, containing 12.5 μL 2 × Taq PCR MasterMix , 3 μL BSA (2 ng/μL), 2 Primer (5 uM), 2 μL template DNA, and 5.5 μL ddH_2_O. Cycling parameters were 95 °C for 5 min, followed by 32 cycles of 95 °C for 45 s, 55 °C for 50 s and 72 °C for 45 s with a final extension at 72 °C for 10 min. The PCR products were purified using a QIAquick Gel Extraction Kit (QIAGEN, Germany), quantified using Real Time PCR, and sequenced at Allwegene Company, Beijing.

Deep sequencing was performed on Miseq platform at Allwegene Company (Beijing). After the run, image analysis, base calling and error estimation were performed using Illumina Analysis Pipeline Version 2.6. The sequencing data have been submitted into the sequence read archive (SRA) database under accession number PRJNA 702646.

For data analysis, qualified reads were separated using the sample-specific barcode sequences and trimmed with Illumina Analysis Pipeline Version 2.6. And then the dataset were analyzed using usearch (version8.1. The sequences were clustered into operational taxonomic units (OTUs) at a similarity level of 97% by uparse method (Edgar 2013), to generate rarefaction curves and to calculate the richness and diversity indices. The rdp Classifier tool (Wang et al. 2007) was used to representative sequences into different taxonomic groups based on the SILVA ribosomal RNA gene database (Quast et al. 2013). Principal coordinates analysis (PCoA) was used to compare groups of samples based on unweighted Uni-Frac distance metrics (Lozupone and Knight 2005), and an unweighted distance-based analysis of molecular variance (AMOVA) was conducted to assess significant differences among samples using the Mothur v.1.3.0 program (Schloss et al. 2009). The similarity between the microbial communities associated with the cotton by-products were analyzed through a hierarchical cluster analysis using the average-neighbor method. This analysis was conducted using the function hclust in the R stats package.

### Functional gene prediction

Phylogenetic investigation of communities by reconstruction of unobserved States (PICRUSt) is a bioinformatics tool that uses 16S ribosomal DNA sequences to predict the functional gene content of microorganisms (Langille et al. 2013). In the present study, we used PICRUSt to obtain an overview of the genomic and metabolic features represented by the adherent bacterial communities in our samples. We associated OTUs with known bacterial genomes precalculated in PICRUSt, by first picking closed OTUs against the Greengenes 16S rRNA gene database (13.5) using QIIME 1.7.0 (Caporaso et al. 2010). The resulting OTU table was then normalized using the script normalize_by_copy_number.py and used for metagenome inference of Kyoto Encyclopedia of Genes and Genomes (KEGG) Orthologs using PICRUSt. The difference in the predicted molecular functions of the bacterial communities attached to the cotton by-products was determined by partial least squares discriminant analysis (PLS-DA) using the SIMCA-P (11.5) software package (Umetrics,Umeå, Sweden).

### Statistical analysis

For comparison between cotton by-products, data for the in situ degradation (animal replicate: N = 3) was subjected to one way analysis of variance. On detection of overall significant differences by analysis of variance, multiple comparisons among least square means were carried out by Duncan’s new multiple range test. The means and standard errors of least square means are reported in the result tables. Significance was declared at *P*<0.05, unless otherwise noted.

We related difference in microbial composition to the chemistry of cottons by-products using redundancy analysis (RDA), as implemented in the Canoco 5.0 software package (Microcomputer Power, Ithaca, NY; Ter Braak and Smilauer 2012). Different chemical composition was introduced as environmental (explanatory) variables. The relative contributions of the top 20 genus-level phylogenetic groups were used as response variables. Redundancy analysis was performed focusing on intersample correlation, and the Monte Carlo Permutation test was applied to decide whether chemical had any statistically significant influence on the microbial composition. Chemical ingredients were considered to have significant effects on microbial composition for *P* < 0.05.

## Results

### Chemical composition and rumen degradation characteristics of cotton by-products

According to the results of chemical analysis of cotton by-products (Additional file 1: Table S1), cottonseed meals presented greater amounts of CP, cottonseed hulls presented greater amounts of NDF and ADF, whereas WCS had greater contents of FG and EE.

Highest in situ ruminal ED of DM, NDF, and ADF were found in CSM, whereas WCS had the highest ED of CP. The degradation of FG mainly happened in the first 6 h, and the disappearance rate of FG in WCS and CSH reached over 90% at 6 h. The maximum disappearance rate of FG among all samples reached over 94% at 72 h, and it ranked as WCS>CSH>CSM (*P*<0.01) (Additional file 1: Table S2).

### Microbial data of 16S rDNA

In the present study, a 16S rDNA gene sequence analysis of samples generated 1,046,756 sequences with a length greater than 200 bp (Additional file 1: Table S3) and an average of 44,510 ± 2025 sequences per sample. Quality filtering by QIIME generated 1,046,578 high-quality reads, accounting for 99% of the raw reads, and an average of 43,607 ± 2209 sequences per sample. The average sequence length was 291 bp.

### Diversity of the bacterial microbiota attached to cotton by-products after 24 h of rumen incubation

The individually based rarefaction curves for each sample (Additional file 1: Fig. S1). and the Good’s coverage which was greater than 0.98 (Additional file 1: Table S3), implying that samples collected at 24 h of rumen incubation provided sufficient OTU coverage for later analysis of bacterial composition. At the 0.03 dissimilarity level, the OTU numbers, Chao 1, and Shannon indices were significantly affected (*P* < 0.01) by the cotton by-products type as shown in Table 1. There was no obvious difference of α diversity between CSH and CSM, whereas the OTU numbers, Chao 1 values and Shannon indices of WCS were significantly lower than CSH and CSM. There was a significant negative correlation between α diversity and the content of EE and FG as shown in Table 2.

**Table 1:** The α diversity of bacterial community attached to cotton by-products after an incubation of 24 h

Item^a^	OTU^b^ numbers	Chao 1 values	Shannon index
CSH	1045^d^	1233^d^	7.5^d^
CSM	1059^d^	1251^d^	7.7^d^
WCS	792^e^	1033^e^	6.3^e^
SEM^c^	18.15	22.87	0.20
*P-*value	< 0.01	< 0.01	< 0.01

^a^CSH, cottonseed hull, including 2 samples; CSM, cottonseed meal, including 3 samples; WCS, whole cottonseed, including 3 samples; each kind of sample was incubated in 3 cows simultaneously

^b^OTU, operational taxonomic units

^c^SEM, standard error of the difference of the means, n = 3

^d,e^Values in a column within the same class without a common superscript are significantly different (*P* < 0.05)

**Table 2:** Pearson correlation coefficients among α diversity of attached bacteria of 24 h and nutritional composition

Item	CP	NDF	ADF	EE	FG
OTU numbers	0.30	− 0.15	− 0.12	− 0.91**	− 0.85**
Chao 1 values	0.30	− 0.16	− 0.12	− 0.89**	− 0.85**
Shannon index	0.28	− 0.16	− 0.14	− 0.69**	− 0.63**

*ADF* acid detergent fiber, *CP* crude protein, *EE* ether extract, *FG* free gossypol, *NDF* neutral detergent fiber, *OTU* operational taxonomic units

^**^P < 0.01

**Fig. 1:**
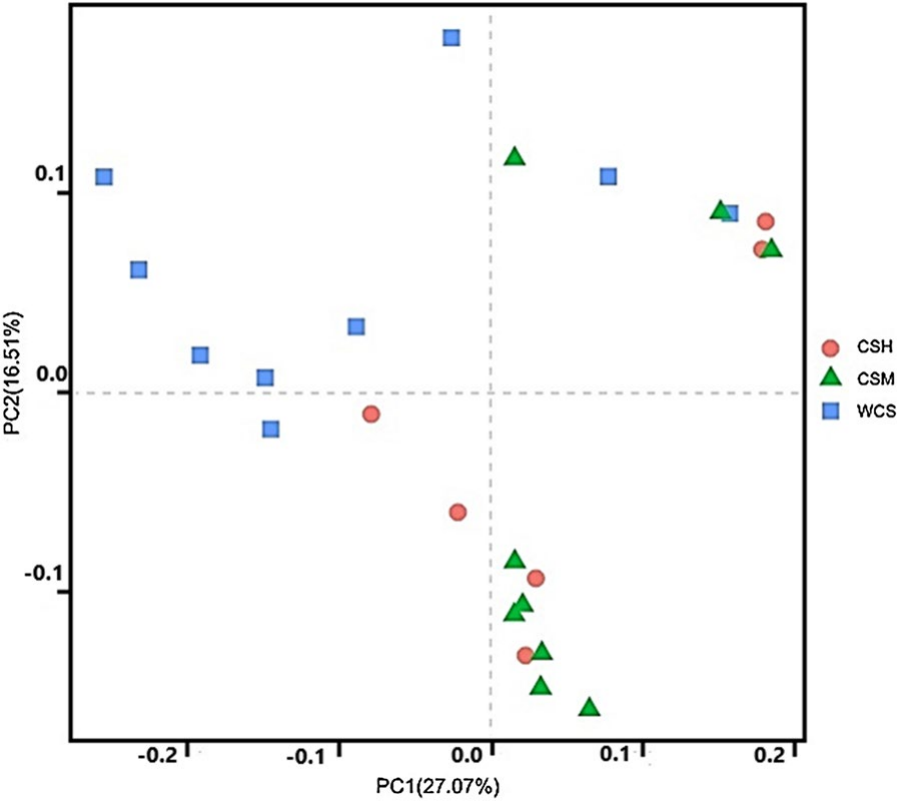
Principal coordinates analysis (PCoA) of microbial diversity across all samples using a unweighted UniFrac metric. The percentage of variation explained by PC1 and PC2 are indicated in the axis. CSH, cottonseed hull; CSM, cottonseed meal; WCS, whole cottonseed

Unweighted UniFrac distance metrics of principal co-ordinates analysis revealed that the attached bacteria composition varied greatly among different cotton by-products (AMOVA: F-statistic = 5.12, *P* <0.01) (Fig. 1).

Hierarchical cluster analysis of the dendrogram showed that 2 clusters were formed, which revealed differences of microbiota composition among rumen incubated cotton by-products (Additional file 1: Fig. S2). Cluster 1 included 2 distinct subclusters. All CSH and CSM samples fell within cluster 1. Conversely, cluster 2 comprised samples of WCS. Subclusters were clearly distinguished by cow, subcluster 1 contained all samples from cow C, and subcluster 2 only contained samples from cow A and cow B.

### Characterization and comparison of the bacterial communities attached to cotton by-products after 24 h of rumen incubation

A total of 21 bacterial phyla were identified among three types of cotton by-products (Additional file 1: Fig. S3). The majority sequences obtained from all cotton by-products belonging to *Bacteroidetes* (CSH, 54.8%, CSM, 58.0%, WCS, 32.6%), *Firmicutes* (CSH, 23.0% , CSM, 26.5%, WCS, 38.4%), *Proteobacteria* (CSH, 12.4%, CSM, 2.9%, WCS, 7.2%), *Cyanobacteria* (CSH, 0.8%, CSM, 0.1%, WCS,14.0%), *Tenericutes* (CSH, 2.3%, CSM, 5.9%, WCS, 1.5%), *Spirochaetae* (CSH, 3.2%, CSM, 1.8%, WCS, 1.2%), and *Verrucomicrobia* (CSH, 1.2%, CSM, 1.9%, WCS, 2.1%) as shown in Table 3. The other 14 phyla were relatively minor (<2% of total sequences) in abundance in comparison.

**Table 3:** Percentage contribution of sequences (%) evaluated at the phyla and genus level across three-kinds of incubated cotton by-products samples of 24 h

Item	CSH	CSM	WCS	SEM^a^	*P*-value
Phyla					
* Bacteroidetes*	54.8^b^	58.0^b^	32.6^c^	2.12	< 0.01
* Firmicutes*	23.0^c^	26.5^c^	38.4^b^	2.94	0.03
* Proteobacteria*	12.4^b^	2.9^c^	7.2^b,c^	1.40	0.02
* Cyanobacteria*	0.8^c^	0.1^c^	14.0^b^	0.78	< 0.01
* Tenericutes*	2.3^c^	5.9^b^	1.5^c^	0.45	< 0.01
* Spirochaetae*	3.2	1.8	1.2	0.83	0.38
* Verrucomicrobia*	1.2	1.9	2.1	0.53	0.55
* Actinobacteria*	0.3	0.5	0.8	0.15	0.20
* SR1_Absconditabacteria*	0.7^b^	0.6^b^	0.3^c^	0.06	0.02
Unidentified	0.3	0.4	0.5	0.09	0.39
* Fibrobacteres*	0.3	0.4	0.1	0.13	0.34
* Euryarchaeota*	0.1	0.3	0.3	0.14	0.55
Other	0.2	0.2	0.3	0.05	0.25
Genus					
* Prevotellaceae UCG-001*	1.2^b^	1.6^b^	0.4^c^	0.07	< 0.01
* Ruminococcaceae UCG-014*	1.2^b^	1.5^b^	0.4^c^	0.18	0.02
* Prevotellaceae UCG-003*	1.2	1.8	0.4	0.36	0.09
* Prevotella 7*	2.0	3.2	1.6	0.70	0.33
* Succinivibrio*	1.7^b^	0.6^c^	0.7^c^	0.13	< 0.01
* Ruminobacter*	1.2^b^	0.2^c^	1.0^b^	0.14	0.01
* Selenomonas 1*	0.9	0.9	1.6	0.30	0.30
* Succinivibrionaceae UCG-002*	2.7^b^	0.8^c^	2.4^b^	0.21	< 0.01
* Rikenellaceae RC9 gut group*	3.6^c^	6.5^b^	0.9^d^	0.52	< 0.01
* Treponema 2*	2.7	1.7	0.9	0.70	0.33
* Succinivibrionaceae UCG-001*	5.4^b^	0.4^c^	1.0^b,c^	1.11	0.07
* Erysipelotrichaceae UCG-002*	2.3^b,c^	1.0^c^	3.8^b^	0.54	0.03
* Succinivibrionaceae UCG-002*	0.3^c^	0.7^c^	2.6^b^	0.47	0.04
* Oribacterium*	0.7	1.9	3.2	0.89	0.28
* Eubacterium coprostanoligenes group*	0.5	0.8	2.3	0.82	0.35
* Lachnoclostridium 1*	0.1	0.9	1.8	0.63	0.31
* Cercis gigantea*	0.7^c^	0.1^c^	14.0^b^	0.79	< 0.01
* Succiniclasticum*	2.1^c^	1.4^c^	9.3^b^	1.54	0.02
Unidentified	23.3^c^	35.9^b^	22.3^c^	1.40	< 0.01
* Prevotella 1*	29.6^b^	18.9^c^	13.7^c^	1.81	< 0.01
Other	15.9	18.5	14.5	1.09	0.12

*CSH* cottonseed hull, including 2 samples, *CSM* cottonseed meal, including 3 samples, *WCS* whole cottonseed, including 3 samples; each kind of sample was incubated in 3 cows simultaneously

^a^*SEM* standard error of the difference of the means, n = 3

^b,c,d^Values in a row within the same class without a common superscript are significantly different (*P* < 0.05)

At the genus level, 178, 162 and 174 taxa were observed among all WCS, CSH and CSM samples, respectively. For clarity and visualization purposes, the top 20 most abundant taxa are presented in Additional file 1: Fig. S4. The most abundant taxa (those with a relative abundance of ≥3%) attached to the rumen-incubated cotton by-product samples included *Prevotella 1*, *Cercis gigantea*, *Rikenellaceae RC9 gut group*, *Prevotella 7*, *Erysipelotrichaceae UCG−002*, and *Succiniclasticum*. The WCS present a greater abundance in *Cercis gigantea* and *Succiniclasticum*, but *Rikenellaceae RC9 gut group* and *Prevotella 1* were greater in CSH and CSM (Table 3).

### Predicted functions of bacteria attached to the cotton by-products after 24 h of rumen incubation

The potential functions of attached bacteria were predicted by using PICRUSt, and a total of 37 gene families were found among all samples (Additional file 1: Table S4; Fig. S5). The majority genes of the obtained 37 gene families were belonged to amino acid metabolism (10.05%), carbohydrate metabolism (10.03%), membrane transport (9.60%), replication and repair (9.58%), energy metabolism (6.71%), and translation (6.28%). Of the 6 predominant gene families mentioned earlier, the relative abundances of the genes involved in membrane transport and energy metabolism were significant greater in WCS samples than CSH and CSM, whereas the abundance of genes involved in amino acid metabolism, carbohydrate metabolism, replication and repair, and translation was significant higher in CSH and CSM than WCS (*P* < 0.01).

### Chemical composition coordinated with microbial colonization

**Fig. 2:**
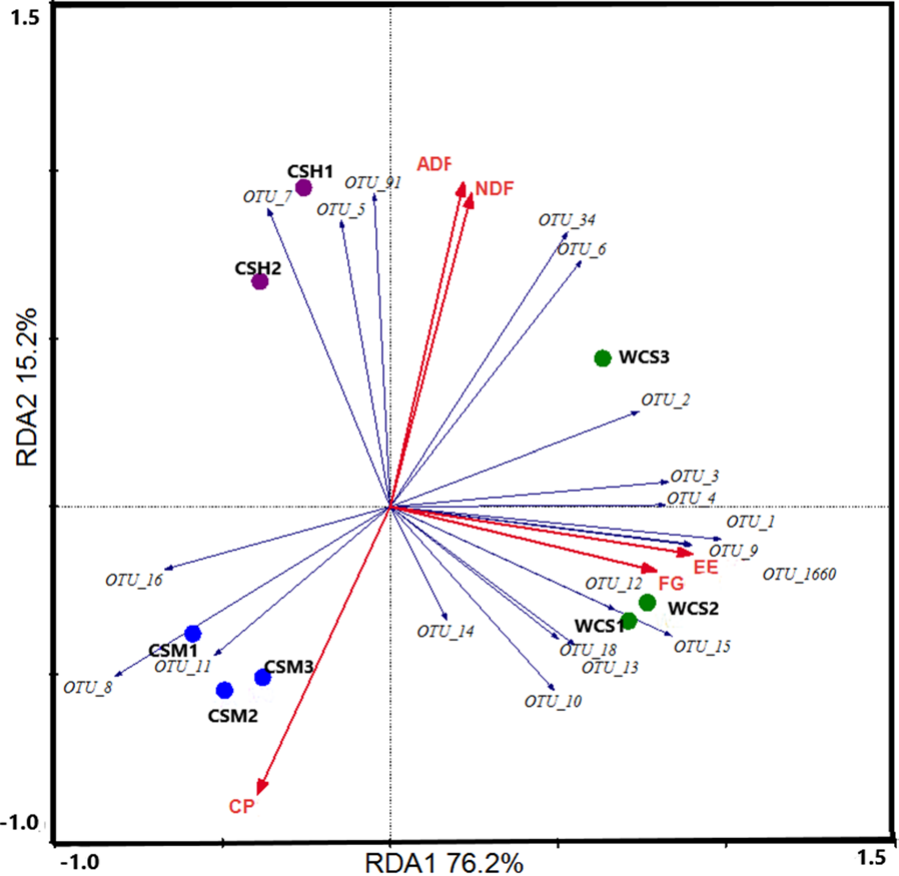
Biplot of the redundancy analysis (RDA) based on the relative abundance of dominant genera (> 1% of total sequence). Constrained explanatory variables are indicated by open triangle arrows. *CSH* cottonseed hull, *CSM* cottonseed meal, *WCS* whole cottonseed, *1–3* sample number of each kind of cotton by-products

We evaluated the effect of the chemical content on the composition of microbiota colonizing the cotton by-products by performing a redundancy analysis using NDF, ADF, EE, CP, and FG as constrained explanatory variables and the relative abundances of dominant genera (those with a relative abundance of ≥2%) as response variables (Fig. 2). Redundancy analysis of the composition of the microbial communities identified the NDF (pseudo-F = 4.4, P = 0.016), ADF (pseudo-F = 2.5, P = 0.076), EE (pseudo-F = 6.4, P = 0.024), CP (pseudo-F =1.3, P = 0.352), and FG (pseudo-F = 2.6, P = 0.036) as significant factors, explaining 76.2 (Axis 1) and 15.2% (Axis 2), respectively, of the variation in microbial composition. On the redundancy analysis ordination plot, FG content was highly correlated with EE content, and the content of NDF, EE and FG content were closely associated with the composition of attached microbiota. 10 genera, including OUT 1 (*Cercis gigantea*), OTU 2 (*Succiniclasticum*), OTU 3 (*Erysipelotrichaceae UCG-002*), OTU 4 (unclassified *Prevotellaceae*), OTU 5 (*Succinivibrionaceae UCG-001*), OTU 6 (*Succinivibrionaceae UCG-002*), OTU 7 (*Prevotella 1*), OTU 9 (unclassified *Bacteroidales S24-7_group*), OTU 34 (*Ruminobacter*), OTU 91 (*Prevotella 1*), were closely correlated with NDF content. In addition, OTU 1, OTU 2, OTU 3, OTU 4, OTU 6, OTU 9, OTU 10 (*Lachnoclostridium 1*), OTU 12 (*Selenomonas 1*), OTU 13 (*Lachnospiraceae*), OTU 14 (unclassified *Verrucomicrobia*), OTU 15 (*Anaerovibrio*), OTU 18 (*Oribacterium*), OTU 34 (*Ruminobacter*), OTU 1660 (*Succiniclasticum*), were closely correlated with EE and FG content.

## Discussion

### Chemical composition and rumen degradation characteristics of cotton by-products

Whole cottonseed, CSH and CSM are important by-products of cotton production in Xinjiang region of China which were widely used in the diet of dairy cattle. The chemical composition of the studied cotton by-products agree with feed tables (Azhdari et al. 2012; Du et al. 2016; Judkins et al. 1991). The greater CP and lower fiber contents seen in WCS and CSM compared to CSH may be explained by the shelling process, which might remove cotton-seed kernel protein, and the shelling and prepressing extraction process lead to a significant lower fat content in CSH and CSM. Due to gossypol is a kind of fat-soluble substance, the content of FG in cotton by-products was closely related with fat content in present study, which was consistent with the result of Gao et al. (2011). The FG content of WCS is mainly affected by varieties, CSM and CSH are by-products obtained from WCS hulling and oil removal, different extraction and pressing methods have different detoxification ability to FG in WCS. Karishma et al. (2016) determined the content range of FG in WCS samples from different sources by HPLC, which varied from 1 to 4 g/kg. In the present experiment, the content of gossypol in WCS2 and WCS3 was consistent with the above results, but FG in WCS1 was greater than the above results, which may be related to WCS varieties and other factors. Wang et al. (2012) reported that the content of FG in CSM was 0.9 g/kg, which was greater than the FG content of CSM samples of present study. The FG content of CSH sample in the present experiment was close to the results of Viana et al. (2015).

**Fig. 3:**
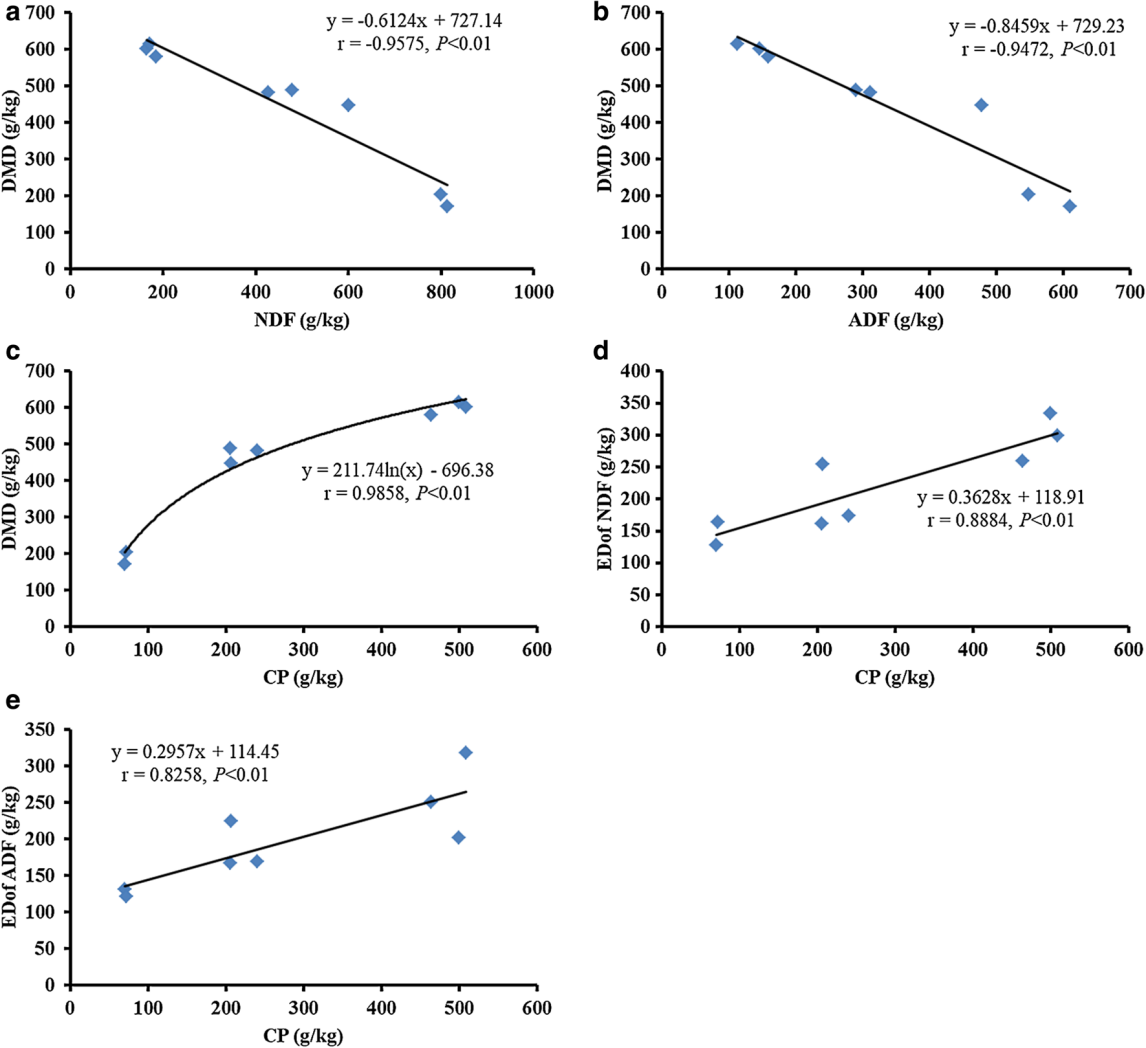
Correlation analysis of chemical composition and rumen effective degradability of cotton by-products. **a** correlation analysis of NDF content and ED of DM, **b** correlation analysis of ADF content and ED of DM, **c** correlation analysis of CP content and ED of DM, **d** correlation analysis of CP content and ED of NDF, **e** correlation analysis of CP content and ED of ADF

Cunha et al. (1998) found that DM degradability with 48 h of incubation time was greater for CSM (62.3 %) followed by broken WCS (57.1 %), CP degradability was also higher for CSM (93.5 %) followed by broken WCS (82.5 %), which was consistent with the results of present study. According to the correlation analysis (Fig. 3), NDF and ADF contents in cotton by-products were negatively correlated with the ED of DM (*P* < 0.01), CP content was positively correlated with the ED degradability of DM (*P* < 0.01), which was consistent with the results of previous study (Kamalak et al. 2005; Kamalak 2006; Mekasha et al. 2002). Additionally, this experiment also demonstrated that CP content was positively correlated with the ED of ADF and NDF (*P* < 0.05), indicating that there was a promoting effect of CP on the degradation of crude fiber in the cotton by-products. In present study, the disappearance rate of gossypol approximately reached above 70% at 0 h, which could be mainly due to the loss caused during the cleaning process of nylon bags. The FG disappearance rate of WCS and CSH reached more than 95% at 6 h, whereas the disappearance rate of FG in CSM increased relatively slow, which reached more than 93% at 36 h. The disappearance rate of FG was above 94% among all samples at 72 h. Generally, the disappearance rate of FG was positive related with its content in cotton by-products. Wang (1995) determined the FG disappearance rate of five WCS samples in an incubation of 24 h by nylon bag test. It was found that the FG disappearance rate of four WCS samples reached over 80% at 1 h, and all WCS samples reached above 97% at 24 h, which were consistent with the results of present study.

The relations of FG content and ED of DM, CP, NDF and ADF were determined by correlation analysis (Table 4). The results showed that FG content in cotton by-products presented a poor correlation with the ED of DM, ADF, NDF and CP. We speculated that this could be due to the detoxification of FG by rumen microorganisms, which weakening the detrimental effect of FG on the digestion and metabolism function of rumen microbes.

**Table 4:** Pearson correlation coefficient of FG content and ED of nutrients

Item	ED			
	DM	CP	NDF	ADF
FG	0.07	0.53	− 0.41	− 0.23

*ADF* acid detergent fiber, *CP* crude protein, *DM* dry matter, *ED* effective degradability, *FG* free gossypol, *NDF* neutral detergent fiber

### Microbial colonization of cotton by-products

Limited information is available on the structure, diversity, and density of attached microbiota of different cotton by-product types. In the present study, results suggesting that cotton by-product types strongly affect the composition and structure of the attached microbiota of rumen incubated samples, as reflected by the clustering of the samples using PCoA and confirmed by AMOVA. We found that more bacteria colonized the CSH and CSM than the WCS, and EE and FG content was significantly negative related with the α diversity of attached bacteria, which could be partly explained by the differing physiochemical conditions of these cotton by-product types, as well as the antibacterial effect of FG.

*Bacteroidetes*, *Firmicutes*, and *Proteobacteria* were the most abundant phyla among all cotton by-products in present study, which was consistent with the result of previous study (Mao et al. 2015). The phylum *Bacteroidetes* was the main degraders of non-fibrous carbohydrates in the rumen (Hopper et al. 2001), and it was significantly more abundant in CSH and CSM samples. Generally, there is a negative correlation of fibre content and the abundant of *Bacteroidetes*. However, the abundant of *Bacteroidetes* was significant lower in WCS than CSH which presented greater content of fibre. In addition, there are large amounts of fibrolytic bacteria in the phylum *Firmicutes* (Evans et al. 2011), and its abundant is positive related with fibre content. However, the phylum *Firmicutes* was significantly more abundant in WCS than CSH and CSM samples, which suggesting that the chemical composition of WCS, including greater content of FG, could inhibit the activity of bacteria in *Bacteroidetes* phylum, and promote the activity of bacteria in *Firmicutes* phylum. During later research, we had demonstrated that gossypol could inhibit the abundance of *Bacteroidetes* bacteria and promote the abundance of *Firmicutes* bacteria by in vitro fermentation using qPCR (Wang et al., 2021).

Among the most abundant taxa at genus level, the present study revealed that the *Cercis gigantea* and *Succiniclasticum* abundance was greater in WCS, *Prevotella 1* and *Rikenellaceae RC9 gut group* was greater in CSH and CSM. At the taxonomic level, *Cercis gigantea* belongs to the phylum of *Cyanobacteria*. To date, there is no report about *Cercis gigantea* in the rumen, but *Cyanobacteria* are common ruminal bacterial phyla and play an important role in nitrate assimilation and reduction of methane production (Prasanna et al. 2002; Flores et al. 2005). *Succiniclasticum* can convert succinic acid into propionic acid, and degrade starch and cellulose. *Prevotella* has been reported as the most abundant genus in the rumen of adult dairy cattle, and it is also associated with ruminal carbohydrate and protein fermentation (Wallace et al. 1999; Chiquette et al. 2008). The present study showed that *Prevotella* spp. dominated in the associated microbiomes of both CSH and CSM, this could explained by the great content of fibre and protein in CSH and CSM, and this finding was consistent with previous report (Stevenson and Weimer 2007). *Rikenellaceae* is a relatively new bacterial family and therefore its metabolic function in the rumen has not yet been defined. Many researchers found that *Rikenellaceae RC9 gut group* relative abundance was negatively affected by the oil content in the diet (Zened et al. 2013; Ramos et al. 2018). The lower relative abundance of *Rikenellaceae RC9 gut group* in WCS may be associated with the greater content of EE.

### Predicted functions of the attached bacteria

With the purpose for further study of the relationship between attached bacteria composition and cotton by-products degradation, the potential functions of the attached bacteria were determined by using PICRUSt to infer putative metagenomes from the 16S rDNA gene profiles. The most abundant functional categories in present study were in agreement with general metabolic functions (such as carbohydrate, protein, and amino acid metabolism) which are essential for the survival of rumen microbiota, and they were also consistent with the observations of previous ruminal metagenomics studies in dairy cows (Parmar et al. 2014; Pitta et al. 2016). Findings of the present study revealed significant differences in bacterial function mainly depending on the chemical composition of cotton by-product samples. Genes relating to energy metabolism were more abundant in the WCS than CSH and CSM, which could be due to the greater content of EE in WCS than CSH and CSM. Yang et al. (2011) found that proteins associated with energy metabolism present a high expression in the detoxification of gossypol by *Aspergillus. Niger*, which noted that greater abundance of genes relating to energy metabolism in WCS could be also due to its greater content of FG. The great abundance of genes related to carbohydrate and amino acids metabolism in CSH and CSM could be due to their greater content of fibre and protein.

Collectively, our results revealed that rumen microbiota presented a high ability of gossypol degradation, and there was a poor correlation of gossypol content and the biodegradation of cotton by-products. Diversity, composition, and potential functions of attached bacteria were significantly affected by cotton by-products types, and the difference of ecological community was closely associated with the chemical composition of incubated cotton by-products, especially NDF, EE, and FG content. These findings are of importance for the targeted improvement of cotton by-products nutrient use efficiency in ruminants and the further research of the detoxification mechanism of gossypol by rumen microbes.

## Supplementary Information

**Additional file 1: Table S1.** Chemical composition of cotton by-products (g/kg DM). **Table S2.** Rumen degradability of DM and nutrients of cotton by-products (%, DM). **Table S3.** Number of sequences, estimated sample coverage, diversity and OTU richness in each sample. **Table S4**. Comparisons of the gene pathways of the bacterial microbiota (% total reads). **Fig S1.** Summary of rarefaction results based on operational taxonomic unit (OTUs) (3% divergence) for each sample.A-C,cows; H, cottonseed hull; M, cottonseed meal; W, whole cottonseed;1-3, sample number of each kind of cottonseed by-products. **Fig S2.** Hierarchical clustering dendrogram representing the OTU table pairwise dissimilarities between the different analyzed samples. OTU, operational taxonomic units; A-C,cows; CSH, cottonseed hull; CSM, cottonseed meal; WCS, whole cottonseed; 1-3, sample number of each kind of cotton by-products. **Fig S3.** Percentage contribution of sequences (%) evaluated at the phylum level across all ruminal-incubated samples.A-C, cows; CSH, cottonseed hull; CSM, cottonseed meal; WCS, whole cottonseed; 1-3, sample number of each kind of cotton by-products. **Fig S4.** Heatmap analyses of 20 most abundant taxa in all samples. The abundance plot shows the proportion of sequences in each sample. A-C, cows; CSH, cottonseed hull; CSM, cottonseed meal; WCS, whole cottonseed; 1-3, sample number of each kind of cotton by-products. **Fig S5.** Variations in the KEGG metabolic pathways in the functional bacterial communities across all rumen-incubated samples. CSH, cottonseed hull; CSM, cottonseed meal; WCS, whole cottonseed; 1-3, sample number of each kind of cotton by-products.

## Data Availability

Data and materials will be made available on reasonable request. Data from the study are available in NCBI-SRA under accession number PRJNA 702646.

## Abbreviations

ADF: Acid detergent fibre
CSH: Cottonseed hull
CSM: Cottonseed meal
CP: Crude protein
DM: Dry matter
EE: Ether extract
ED: Effective degradability
FG: Free gossypol
HPLC: High-performance liquid chromatography
KEGG: Kyoto Encyclopedia of Genes and Genomes
NDF: Neutral detergent fibre
OTU: Operational taxonomic units
PCoA: Principal coordinates analysis
RDA: Redundancy analysis
WCS: Whole cottonseed
