# Supplementary material for: In situ rumen degradation characteristics and bacterial colonization of whole cottonseed, cottonseed hull and cottonseed meal with different gossypol content
Source: AMB Express. 2021 Jun 22;11:91. doi: 10.1186/s13568-021-01244-2 (PMC8218095; doi:10.1186/s13568-021-01244-2)
Supplement: Supplementary file 1 — Additional file 1: Table S1. Chemical composition of cotton by-products (g/kg DM). Table S2. Rumen degradability of DM and nutrients of cotton by-products (%, DM). Table S3. Number of sequences, estimated sample coverage, diversity and OTU richness in each sample. Table S4. Comparisons of the gene pathways of the bacterial microbiota (% total reads). Fig S1. Summary of rarefaction results based on operational taxonomic unit (OTUs) (3% divergence) for each sample.A-C,cows; H, cottonseed hull; M, cottonseed meal; W, whole cottonseed;1-3, sample number of each kind of cottonseed by-products. Fig S2. Hierarchical clustering dendrogram representing the OTU table pairwise dissimilarities between the different analyzed samples. OTU, operational taxonomic units; A-C,cows; CSH, cottonseed hull; CSM, cottonseed meal; WCS, whole cottonseed; 1-3, sample number of each kind of cotton by-products. Fig S3. Percentage contribution of sequences (%) evaluated at the phylum level across all ruminal-incubated samples.A-C, cows; CSH, cottonseed hull; CSM, cottonseed meal; WCS, whole cottonseed; 1-3, sample number of each kind of cotton by-products. Fig S4. Heatmap analyses of 20 most abundant taxa in all samples. The abundance plot shows the proportion of sequences in each sample. A-C, cows; CSH, cottonseed hull; CSM, cottonseed meal; WCS, whole cottonseed; 1-3, sample number of each kind of cotton by-products. Fig S5. Variations in the KEGG metabolic pathways in the functional bacterial communities across all rumen-incubated samples. CSH, cottonseed hull; CSM, cottonseed meal; WCS, whole cottonseed; 1-3, sample number of each kind of cotton by-products. [file 13568_2021_1244_MOESM1_ESM.pdf]

AMB express

In situ rumen degradation characteristics and bacterial colonization of whole cottonseed, cottonseed hull and cottonseed meal with different gossypol content

Weikang Wang[1], Yanlu Wang[1], Wenjuan Li[1], Qichao Wu[1], Kailun Yang[2], Shengli Li[1], Hongjian Yang[1,*]

[1]State Key Laboratory of Animal Nutrition, College of Animal Science and Technology, China Agricultural University, Beijing 100193, China

[2]College of Animal Sciences, Xinjiang Agricultural University, Urumuqi 830052, China

*** Corresponding Author**, Hong-Jian Yang, E-mail: yang_hongjian@sina.com

Supplemental Table S1. Chemical composition of cotton by-products (g/kg DM)

Item[1]	NDF[2]	ADF[2]	CP[2]	EE[2]	FG[2]
CSH1	813[a]	610[a]	70[d]	28[d]	0.77[d]
CSH2	799[a]	548[ab]	72[d]	20[d]	0.39[f]
CSM1	164[d]	146[d]	508[a]	11[d]	0.55[e]
CSM2	171[d]	112[d]	499[a]	19[d]	0.21[fg]
CSM3	184[d]	159[d]	463[b]	7[d]	0.07[g]
WCS1	478[c]	289[c]	206[c]	233[a]	8.39[a]
WCS2	427[c]	311[c]	240[c]	134[b]	3.16[b]
WCS3	600[b]	477[b]	206[c]	108[c]	2.42[c]
SEM[3]	65.2	47.0	44.2	19.7	0.68
P-value	<0.01	<0.01	<0.01	<0.01	<0.01

[1]CSH, cottonseed hull; CSM, cottonseed meal; WCS, whole cottonseed; 1-3, sample number of each kind of cotton by-products.

[2]ADF, acid detergent fiber; CP, crude protein; EE, ether extract; FG, free gossypol; NDF, neutral detergent fiber.

[3]SEM, standard error of the difference of the means, n = 8.

[a,b,c,d,e,f,g]Values in a column within the same class without a common superscript are significantly different (P<0.05).

Supplemental Table S2. Rumen degradability of DM and nutrients of cotton by-products (%, DM)

Item[1]	Incubation time							Degradation kinetics[2]			
	0 h	6 h	12 h	24 h	36 h	48 h	72 h	a (%)	b (%)	c (%/h)	ED(%)
**DM**											
CSH	11.6^{c}	16.1^{c}	17.1^{c}	23.6^{c}	24.7^{c}	27.9^{c}	37.4^{c}	12.1^{b}	44.3^{ab}	1.0^{b}	18.4^{c}
CSM	25.9^{b}	55.6^{a}	58.5^{a}	73.3^{a}	81.0^{a}	81.8^{a}	83.7^{a}	29.4^{a}	53.8a	8.0^{a}	59.8^{a}
WCS	28.2^{a}	43.4^{b}	51.9^{b}	55.3^{b}	60.1^{b}	61.7^{b}	64.5^{b}	30.9^{a}	36.2^{b}	7.4^{a}	47.5^{b}
SEM[3]	0.51	0. 65	1.87	1.3.7	1.27	1.14	2.09	1.28	2.99	0.82	0.81
P-value	<0.01	<0.01	<0.01	<0.01	<0.01	<0.01	<0.01	<0.01	<0.01	<0.01	<0.01
**CP**											
CSH	42.3^{b}	37.9^{c}	42.1^{c}	43.7^{c}	45.5^{b}	47.9^{b}	48.3^{c}	39.9^{b}	37.9^{b}	3.3^{b}	43.2^{c}
CSM	15.2^{c}	55.2^{b}	68.3^{b}	82.1^{b}	91.9^{a}	89.5^{a}	94.7^{a}	17.2^{c}	75.0^{a}	9.9^{a}	63.9^{b}
WCS	47.7^{a}	67.5^{a}	83.7^{a}	87.2^{a}	89.9^{a}	87.5^{a}	89.1^{b}	48.5^{a}	41.8^{b}	10.0^{a}	74.6^{a}
SEM[3]	1.68	1.36	1.13	1.05	1.13	1.04	0.84	1.46	1.45	0.76	0.72
P-value	<0.01	<0.01	<0.01	<0.01	<0.01	<0.01	<0.01	<0.01	<0.01	<0.01	<0.01
**NDF**											
CSH	2.8^{b}	4.0^{c}	6.7^{b}	15.7^{b}	19.1^{b}	19.7^{c}	33.0^{b}	10.0^{b}	35.9	1.1^{b}	14.6^{b}
CSM	7.5^{a}	22.9^{a}	23.8^{a}	36.1^{a}	41.0^{a}	49.6^{a}	53.8^{a}	13.1^{a}	46.4	3.7^{ab}	29.7^{a}
WCS	7.1^{a}	15.6^{b}	25.9^{a}	27.8^{a}	35.3^{a}	40.3^{b}	42.5^{ab}	10.8^{ab}	41.7	3.9^{a}	20.0^{b}
SEM[3]	0.87	1.63	2.40	3.04	3.14	2.60	3.45	0.93	4.97	0.83	2.28

P-value	<0.01	<0.01	<0.01	<0.01	<0.01	<0.01	<0.05	0.10	0.45	0.09	<0.01
**ADF**											
CSH	4.3^{b}	10.5^{b}	13.6^{b}	15.5^{b}	19.2^{b}	20.5^{b}	25.4^{b}	4.6^{b}	22.8	4.9	12.6^{c}
CSM	13.0^{a}	19.6^{a}	29.5^{a}	32.3^{a}	34.9^{a}	37.9^{a}	45.5^{a}	16.2^{a}	30.5	5.1	25.7^{a}
WCS	3.5^{b}	17.5^{a}	21.5^{a}	23.7^{b}	28.6^{a}	31.1^{a}	36.6^{ab}	7.0^{b}	33.2	6.4	18.9^{b}
SEM[3]	1.66	1.68	1.92	2.40	2.35	2.94	4.39	1.45	5.99	1.20	1.81
P-value	<0.01	<0.05	<0.01	<0.01	<0.01	<0.01	0.05	<0.01	0.56	0.68	<0.01
**FG**											
CSH	69.3^{b}	95.4^{a}	96.8^{a}	98.1^{a}	98.4^{a}	98.4^{a}	98.5^{a}				
CSM	70.5^{b}	81.8^{b}	84.8^{b}	89.4^{b}	93.0^{b}	93.7^{b}	94.1^{b}				
WCS	79.4^{a}	96.2^{a}	97.5^{a}	98.8^{a}	99.0^{a}	99.0^{a}	99.1^{a}				
SEM[3]	1.77	3.30	2.11	1.43	1.06	0.98	0.92				
P-value	<0.01	<0.05	<0.01	<0.01	<0.01	<0.01	<0.01				

[1]CSH, cottonseed hull, including 2 samples; CSM, cottonseed meal, including 3 samples; WCS, whole cottonseed, including 3 samples; each kind of sample was incubated in 3 cows simultaneously; ADF, acid detergent fiber; CP, crude protein; DM, dry matter; FG, free gossypol; NDF, neutral detergent fiber.

[2] a, soluble and very rapidly degradable component; b, insoluble, but potentially degradable component; c, a constant degradation rate per unit time of b; ED, effective degradability.

[3]SEM, standard error of the difference of the means, n = 3.

[a,b,c]Values in a column within the same class without a common superscript are significantly different (*P*<0.05).

Supplemental Table S3. Number of sequences, estimated sample coverage, diversity and OTU richness in each sample

Sampling type	Sample ID[1]	Sequences	OTU[2]	Chao1	Coverage	Shannon
Cottonseed hull	A-CSH1	44493	841	1034	0.99	6.18
	A-CSH2	45515	970	1203	0.99	7.56
	B-CSH1	45994	1095	1259	0.99	7.98
	B-CSH2	42810	1060	1248	0.99	7.62
	C-CSH1	44886	1032	1235	0.99	7.69
	C-CSH2	47079	1072	1222	0.99	8.01
Cottonseed meal	A-CSM1	41867	1063	1255	0.99	7.77
	A-CSM2	44746	1111	1346	0.98	7.98
	A-CSM3	46650	1045	1284	0.99	7.77
	B-CSM1	46301	1046	1205	0.99	7.74
	B-CSM2	42887	1093	1273	0.99	7.81
	B-CSM3	45482	1103	1290	0.99	7.89
	C-CSM1	40593	826	1004	0.99	7.19
	C-CSM2	42474	1037	1206	0.99	7.91
	C-CSM3	40808	978	1156	0.99	7.54
Whole cottonseed	A-WCS1	42833	763	969	0.99	6.40
	A-WCS2	43056	672	849	0.99	5.28
	A-WCS3	46425	808	1036	0.99	5.94
	B-WCS1	43916	713	942	0.99	5.39
	B-WCS2	44902	827	1061	0.99	5.82
	B-WCS3	46707	891	1116	0.99	6.45
	C-WCS1	43449	928	1137	0.99	7.08
	C-WCS2	47684	755	948	0.99	7.01
	C-WCS3	46697	1003	1162	0.99	7.74

[1]A-C, cows; CSH, cottonseed hull; CSM, cottonseed meal; WCS, whole cottonseed; 1-3, sample number of each kind of cotton by-products.

[2]OTU, operational taxonomic units.

Supplemental Table S4. Comparisons of the gene pathways of the bacterial microbiota (% total reads).

Item[1]	CSH	CSM	WCS	SEM[2]	*P*-value
Xenobiotics Biodegradation and Metabolism	1.4[b]	1.3[c]	1.7[a]	0.01	<0.01
Membrane Transport	9.0[b]	9.0[b]	10.5[a]	0.24	0.01
Endocrine System	0.4[a]	0.3[b]	0.4[a]	0.01	0.03
Infectious Diseases	0.4[b]	0.3[c]	0.5[a]	0.01	<0.01
Amino Acid Metabolism	10.2[a]	10.3[a]	9.6[b]	0.06	<0.01
Excretory System	0.02[a]	0.02[a]	0.02[a]	0.00	0.50
Neurodegenerative Diseases	0.1[a]	0.1[a]	0.1[a]	0.00	0.01
Metabolism	2.6[a]	2.5[b]	2.6[a]	0.01	<0.01
Carbohydrate Metabolism	10.2[a]	10.2[a]	9.6[b]	0.06	<0.01
Translation	6.4[b]	6.6[a]	5.7[c]	0.04	<0.01
Immune System	0.1[a]	0.1[a]	0.08[b]	0.00	<0.01
Cell Growth and Death	0.6[a]	0.6[a]	0.6[a]	0.01	0.97
Lipid Metabolism	2.6[a]	2.6[a]	2.6[a]	0.02	0.45
Cell Motility	1.7[a]	1.9[a]	1.9[a]	0.11	0.43
Signaling Molecules and Interaction	0.2[a]	0.2[a]	0.2[a]	0.00	0.36
Transcription	2.3[a]	2.3[a]	2.1[b]	0.02	0.02
Replication and Repair	9.9[a]	10.0[a]	8.9[b]	0.05	<0.01
Biosynthesis of Other Secondary Metabolites	1.0[a]	1.0[a]	1.0[a]	0.01	0.18
Digestive System	0.08[a]	0.07[b]	0.08[a]	0.00	<0.01
Metabolism of Terpenoids and Polyketides	1.7[b]	1.7[b]	1.8[a]	0.01	<0.01
Metabolism of Cofactors and Vitamins	4.7[b]	4.6[b]	4.9[a]	0.05	0.01
Cancers	0.1[a]	0.09[b]	0.1[a]	0.00	<0.01
Signal Transduction	1.2[b]	1.2[b]	1.6[a]	0.01	<0.01
Energy Metabolism	6.2[b]	6.2[b]	7.4[a]	0.07	<0.01
Cellular Processes and Signaling	3.9[a]	3.7[b]	3.6[b]	0.03	0.03
Folding, Sorting and Degradation	2.7[a]	2.7[a]	2.6[a]	0.01	0.14
Nervous System	0.1[a]	0.1[a]	0.1[a]	0.00	0.05
Environmental Adaptation	0.1[ab]	0.2[a]	0.09[b]	0.00	0.02
Metabolism of Other Amino Acids	1.6[a]	1.6[a]	1.6[a]	0.01	0.29
Enzyme Families	2.2[b]	2.2[b]	2.4[a]	0.01	<0.01
Transport and Catabolism	0.3[b]	0.4[a]	0.2[c]	0.01	<0.01
Poorly Characterized	4.8[b]	4.7[b]	5.0[a]	0.02	<0.01
Glycan Biosynthesis and Metabolism	3.0[a]	2.9[a]	2.4[b]	0.06	<0.01
Immune System Diseases	0.05[a]	0.05[a]	0.04[b]	0.00	<0.01
Genetic Information Processing	2.5[b]	2.5[b]	2.7[a]	0.01	<0.01
Nucleotide Metabolism	4.4[a]	4.5[a]	3.9[b]	0.02	<0.01
Metabolic Diseases	0.1[b]	0.1[b]	0.2[a]	0.00	0.01

[1]CSH, cottonseed hull, including 2 samples; CSM, cottonseed meal, including 3 samples; WCS, whole cottonseed, including 3 samples; each kind of sample was incubated in 3 cows simultaneously.

[2]SEM, standard error of the difference of the means, n =3 .
[a,b,c] Values in a row within the same class without a common superscript are significantly different (P<0.05).

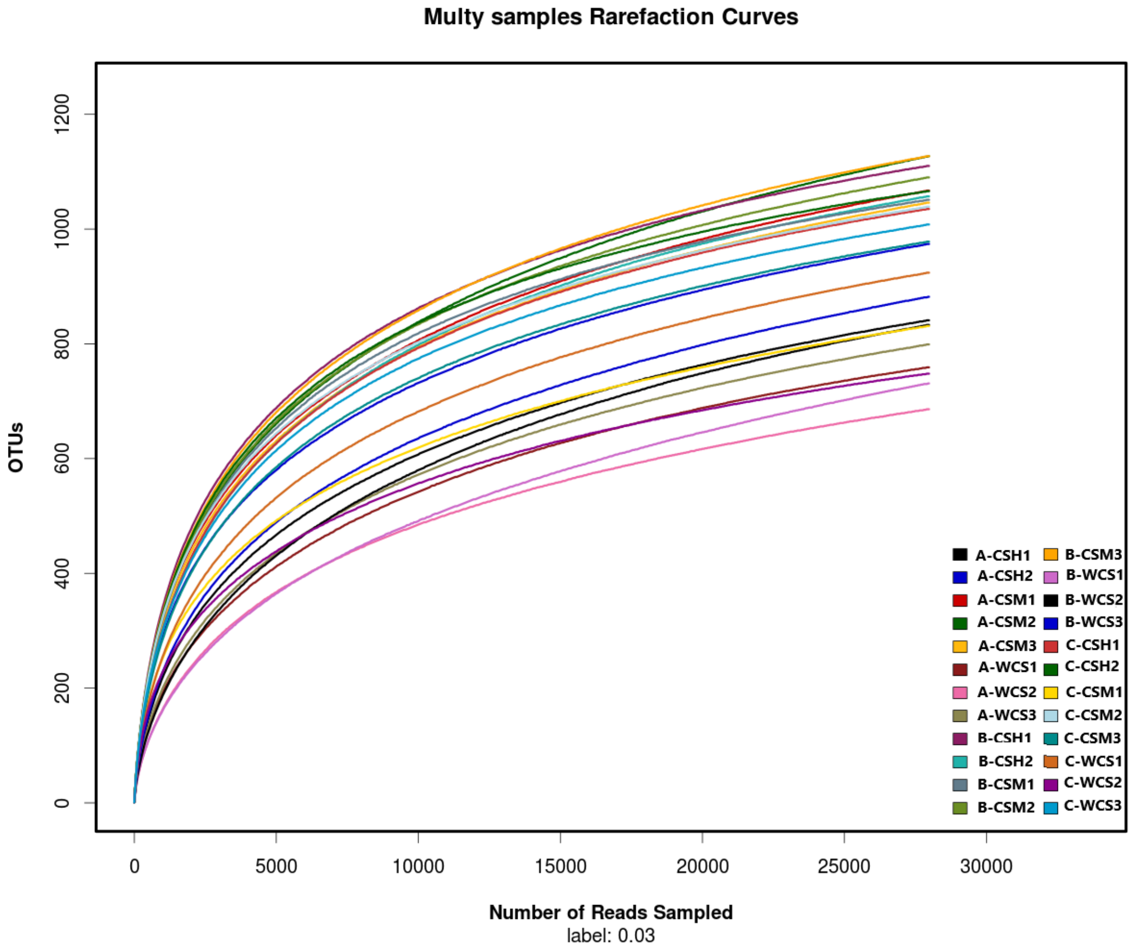

Supplemental Fig S1. Summary of rarefaction results based on operational taxonomic unit (OTUs) (3% divergence) for each sample. A-C, cows; H, cottonseed hull; M, cottonseed meal; W, whole cottonseed; 1-3, sample number of each kind of cottonseed by-products.

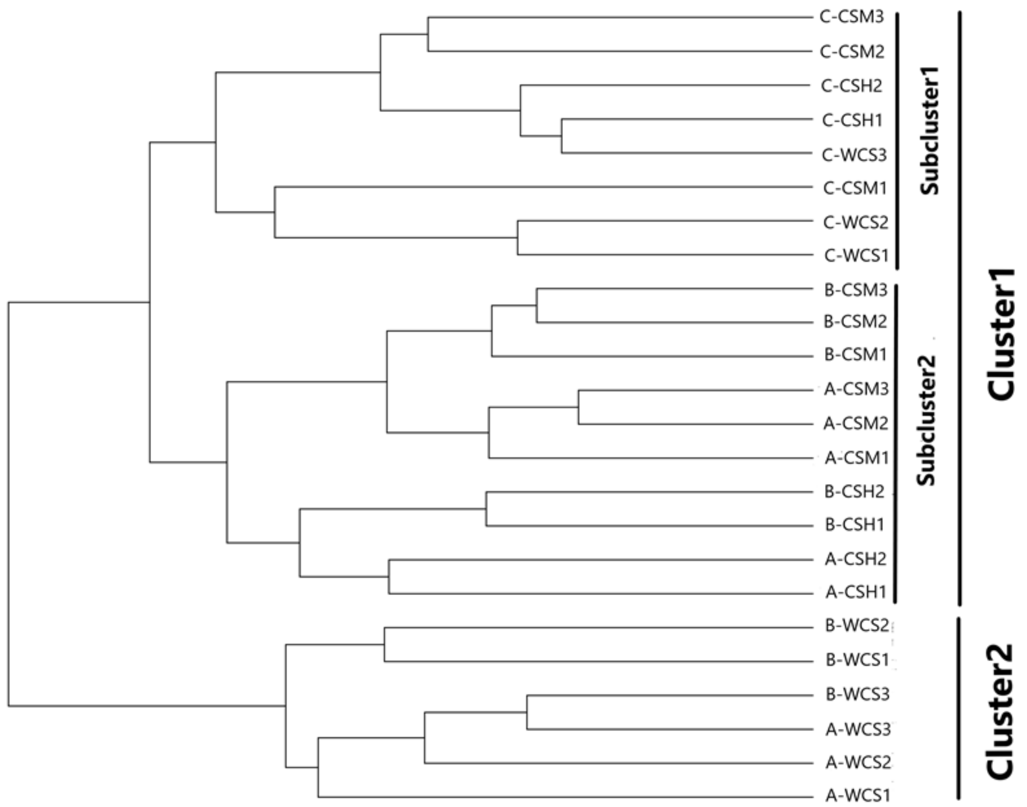

Supplemental Fig S2. **Hierarchical clustering dendrogram representing the OTU table pairwise dissimilarities between the different analyzed samples. OTU, operational taxonomic units; A-C, cows; CSH, cottonseed hull; CSM, cottonseed meal; WCS, whole cottonseed; 1-3, sample number of each kind of cotton by-products.**

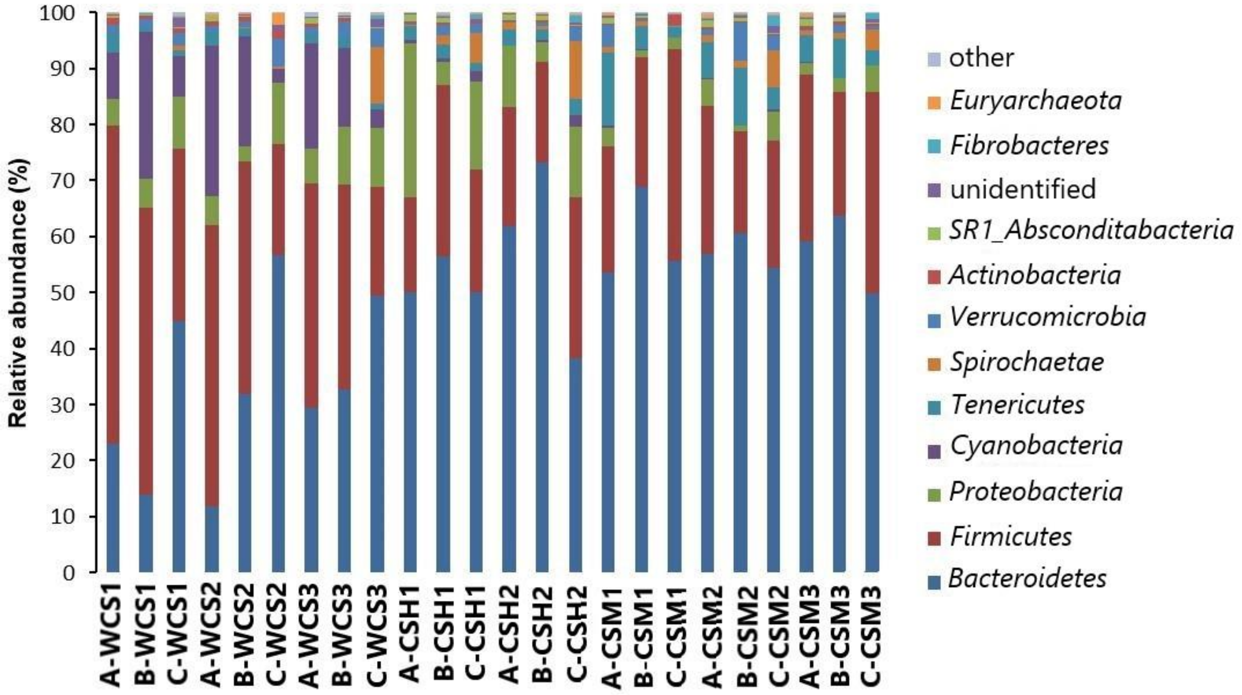

Supplemental Fig S3. **Percentage contribution of sequences (%) evaluated at the phylum level across all ruminal-incubated samples. A-C, cows; CSH, cottonseed hull; CSM, cottonseed meal; WCS, whole cottonseed; 1-3, sample number of each kind of cotton by-products.**

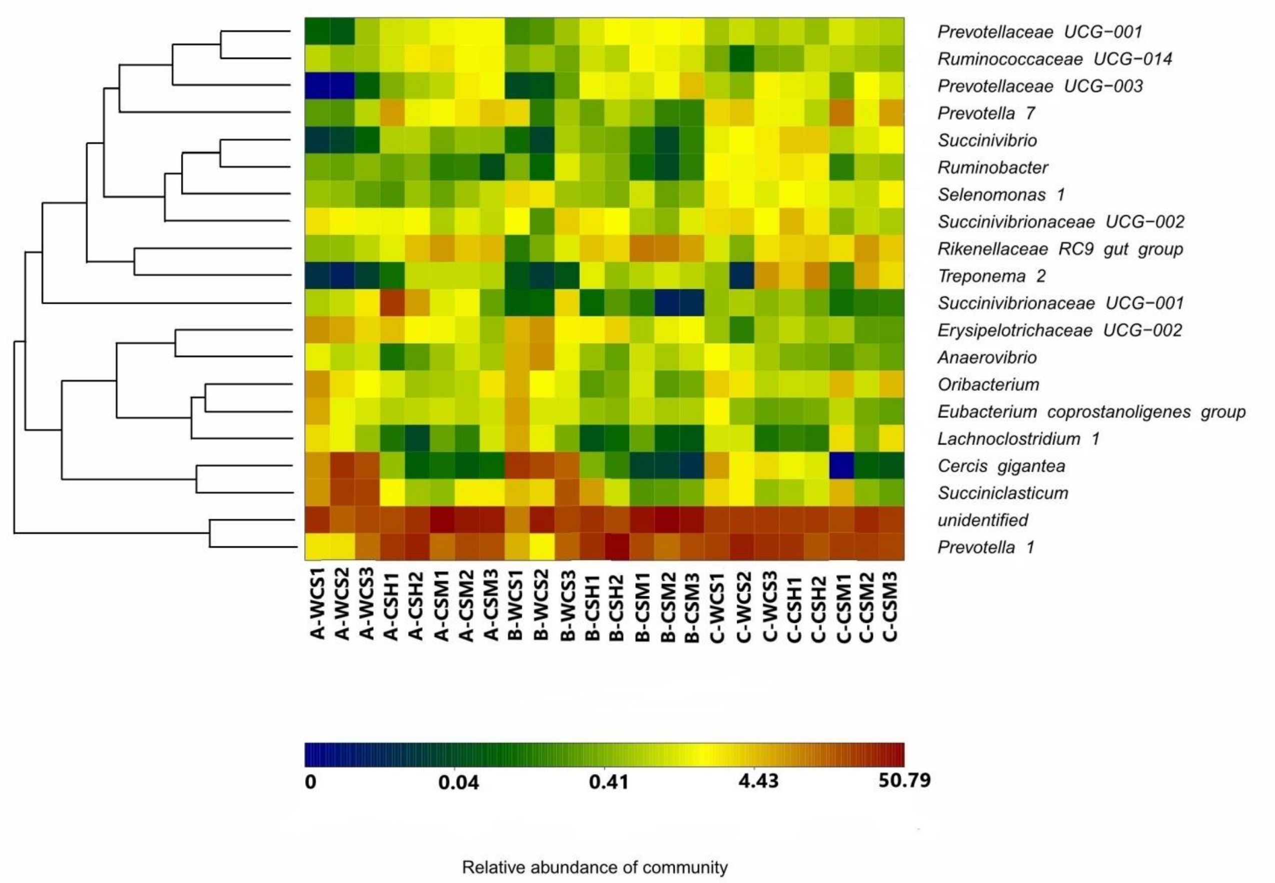

Supplemental Fig S4. **Heatmap analyses of 20 most abundant taxa in all samples. The abundance plot shows the proportion of sequences in each sample. A-C, cows; CSH, cottonseed hull; CSM, cottonseed meal; WCS, whole cottonseed; 1-3, sample number of each kind of cotton by-products.**

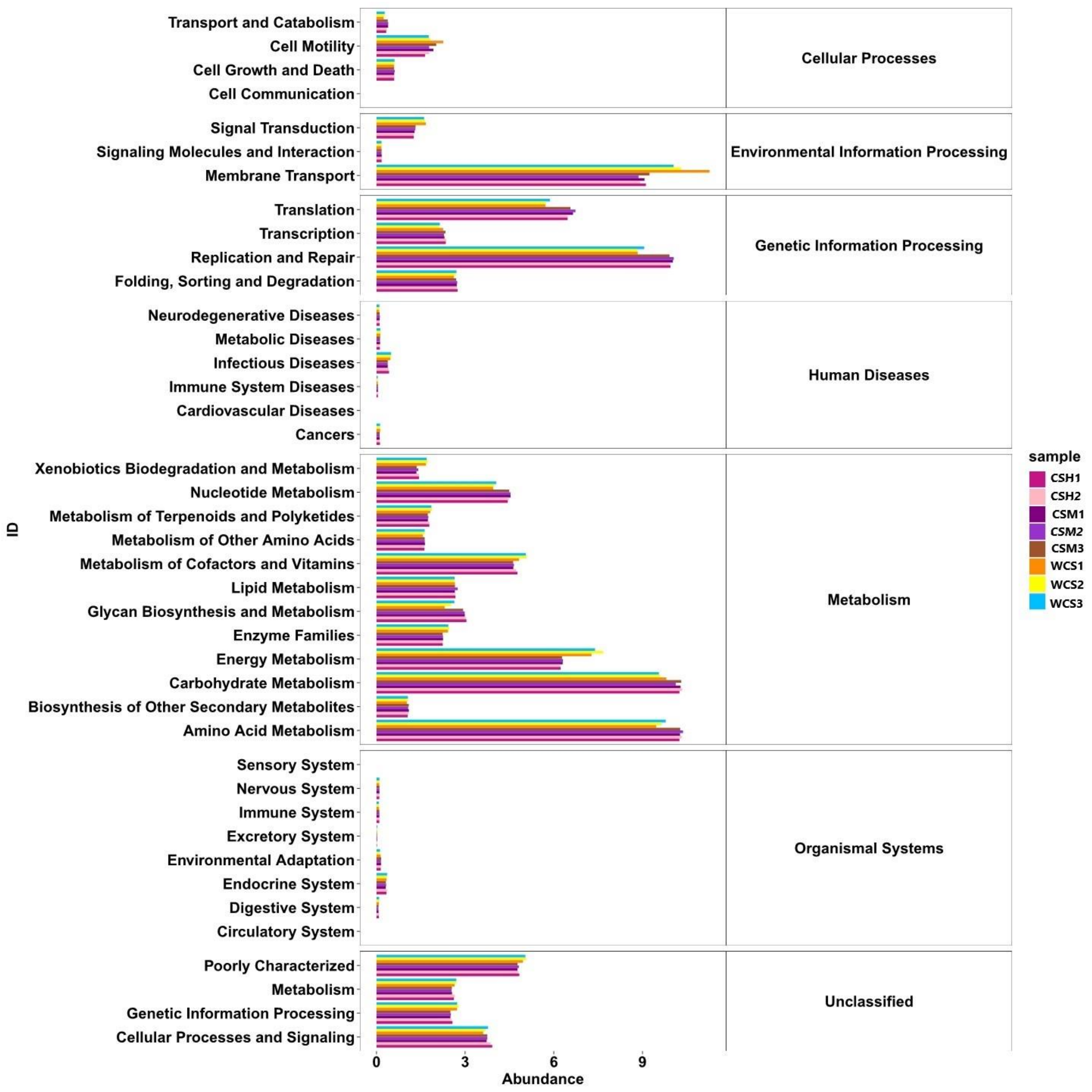

Supplemental Fig S5. Variations in the KEGG metabolic pathways in the functional bacterial communities across all rumen-incubated samples. CSH, cottonseed hull; CSM, cottonseed meal; WCS, whole cottonseed; 1-3, sample number of each kind of cotton by-products.
